# Characterisation of sub-clinical primary myocardial disease in systemic sclerosis - preliminary findings from a cardiac magnetic resonance study

**DOI:** 10.1186/1532-429X-16-S1-P335

**Published:** 2014-01-16

**Authors:** Bara Erhayiem, Lesley-Anne Bissell, Adam K McDiarmid, Ananth Kidambi, David P Ripley, John P Greenwood, Steven Sourbron, Francesco Del Galdo, Paul Emery, Jacqueline Andrews, Maya Buch, Sven Plein

**Affiliations:** 1 Multidisciplinary Cardiovascular Research Centre & The Division of Cardiovascular and Diabetes Research, Leeds Institute of Genetics, Health & Therapeutics, University of Leeds, Leeds, West Yorkshire, UK; 2 Leeds Institute of Rheumatic and Musculoskeletal Medicine, University of Leeds, Leeds, West Yorkshire, UK; 3 Department of Medical Physics, University of Leeds, Leeds, West Yorkshire, UK

## Background

Systemic sclerosis (SSc) is a chronic disease characterised by systemic inflammation, vasculopathy and fibrosis. Primary myocardial disease occurs in both limited (lcSSc) and diffuse (dcSSc) cutaneous subtypes, and carries a poor prognosis. The natural history is poorly understood, with no clear approach to identifying the 'at-risk' patient. CMR studies in SSc have rarely correlated with disease phenotype. Our objective is to determine cardiovascular manifestations of SSc using multi-component CMR.

## Methods

Eleven patients fulfilling SSc ACR/Le-Roy criteria, without known CV disease or diabetes, underwent CMR at 3.0T (Philips Achieva TX). Data from 10 healthy subjects served as a control group. Standard bSSFP cine images were acquired and LV dimensions calculated. First-pass perfusion imaging in three short-axis LV slices was performed during administration of 0.1 mmol/kg of gadobutrol at 3 minutes of 140 mcg/kg/min adenosine for stress and repeated 15 minutes later at rest. Myocardial perfusion reserve (MPR) was calculated using Fermi deconvolution (PMI v.0.4, [Sourbron, 2009] with basal blood pool providing the arterial input. Native and 15 minute post final contrast T1 maps were generated from mid-LV short axis using a modified 3,3,5 Look-Locker inversion sequence to calculate extra-cellular volume (ECV) fraction.

## Results

Patient characteristics were as follows: Mean age 54 ± 16, disease duration 9.6 ± 7.3 years, 7 female, 7 lcSSc subtype, 4 dcSSc subtype, 5 interstitial lung disease. Smoking status: 1 current, 8 ex-smoker, 2 never. 3 have known hypertension. CMR measurements are presented in Table 1. One patient with lsSSc subtype showed lateral mid-ventricular wall enhancement on LGE. ECV increased and MPR decreased with age (r = 0.657, p = 0.039; rho= -0.75, p = 0.02 respectively). A non-significant trend for a higher ECV was noted in lcSSc subtype (mean ECV 33% vs. 28% dcSSc subtype, p = 0.14). ECV was similar for disease duration (mean ECV 33% > 10 years vs. 30% < 10 years, p = 0.2). MPR was non-significantly lower in lcSSc subtype (mean 1.83 vs. 2.13 in dcSSc subtype, p = 0.3) and there was no strong association with disease duration. MPR in SSc was lower than in healthy controls (n = 9) (1.9 versus 2.8, p = 0.03).

**Table 1:** CMR measurements in eleven patients with systemic sclerosis

Variable	Mean (SD)
End-diastolic volume index (ml/m2)	85 (19)

End-systolic volume index (ml/m2)	32 (10)

Stroke volume index (ml/m2)	53 (11)

LV ejection fraction index (%/m2)	38 (6)

LV mass index (g/m2)	46 (9)

Extra-cellular volume fraction (%)	32 (5)

Myocardial perfusion reserve	1.9 (0.4)

## Conclusions

CMR demonstrates lower MPR in patients with SSc than in controls. Our preliminary data suggest that quantitative CMR may also be able to detect differences in myocardial involvement in subtypes of the disease, justifying future larger studies.

## Funding

1. Raynaud's and scleroderma association. 2. IMID cardiovascular outcomes program grant, Schering Plough.

